# MAW point mutation impairs *H*. *Seropedicae* RecA ATP hydrolysis and DNA repair without inducing large conformational changes in its structure

**DOI:** 10.1371/journal.pone.0214601

**Published:** 2019-04-18

**Authors:** Wellington C. Leite, Renato F. Penteado, Fernando Gomes, Jorge Iulek, Rafael M. Etto, Sérgio C. Saab, Maria B. R. Steffens, Carolina W. Galvão

**Affiliations:** 1 Department of Physics, State University of Ponta Grossa (UEPG), Ponta Grossa,Paraná, Brazil; 2 Department of Chemistry, State University of Ponta Grossa (UEPG), Ponta Grossa, Paraná, Brazil; 3 Department of Genetics and Evolutionary Biology, Institute of Biosciences, University of São Paulo, São Paulo, São Paulo, Brazil; 4 Department of Biochemistry and Molecular Biology, Federal University of Paraná, Curitiba, Paraná, Brazil; 5 Department of Structural and Molecular Biology and Genetics, State University of Ponta Grossa (UEPG), Ponta Grossa, Paraná, Brazil; Istituto di Genetica Molecolare, ITALY

## Abstract

RecA is a multifunctional protein that plays a central role in DNA repair in bacteria. The structural Make ATP Work motif (MAW) is proposed to control the ATPase activity of RecA. In the present work, we report the biochemical activity and structural effects of the L53Q mutation at the MAW motif of the RecA protein from *H*. *seropedicae* (HsRecA L53Q). *In vitro* studies showed that HsRecA L53Q can bind ADP, ATP, and ssDNA, as does wild-type RecA. However, the ATPase and DNA-strand exchange activities were completely lost. *In vivo* studies showed that the expression of HsRecA L53Q in *E*. *coli recA*1 does not change its phenotype when cells were challenged with MMS and UV. Molecular dynamics simulations showed the L53Q point mutation did not cause large conformational changes in the HsRecA structure. However, there is a difference on dynamical cross-correlation movements of the residues involved in contacts within the ATP binding site and regions that hold the DNA binding sites. Additionally, a new hydrogen bond, formed between Q53 and T49, was hypothesized to allow an independent motion of the MAW motif from the hydrophobic core, what could explain the observed loss of activity of HsRecA L53Q.

## 1. Introduction

The bacterial RecA protein plays a central role in (1) the repair of stalled replication forks and double-strand break, (2) general recombination, (3) induction of the SOS response, and (4) translesion DNA synthesis [1,2]. Its functions *in vivo* depend upon several key activities identified *in vitro*, including the binding of DNA molecules and the hydrolysis of ATP. RecA L1 and L2 motifs form the DNA binding sites and RecA Walker A and B motifs, the ATP (or other NTPs) binding and hydrolysis site [3,4]. The residues of the Walker A Motif (or P-loop) in the *E*. *coli* protein are 66-GPESSGKTT-73, while of the Walker B Motif are 140-VIVVD-144 [5]. Together with the Make ATP Work motif (MAW) (*E*. *coli* residues, 42-TGSLSLDIALGAGGLPMGRIVEIY-65), these three motifs have been shown to be important for RecA to adopt its high-affinity conformation for ssDNA binding [6,7]. They comprise the most conserved part of RecA homologues in virtually all organisms [8]. The MAW motif consists of a loop, an α-helix, a tight turn, and of a final β-strand, rich in glycine, which threads through the RecA hydrophobic core that is a part of the ATPase domain [9]. However, the MAW motif itself has not been shown to directly contact the cofactors ADP, ATP and others NTPs [6,10–15]. It also connects the Walker A motif to a hinge that undergoes a dramatic change in the transition from inactive to active RecA conformation, therefore, it has been proposed to be a modulator of interactions with DNA and to act as a conformation switch that responds to different cofactors [8,9,16]. However, this motif, despite being conserved in the RecA family of proteins, remains relatively unexplored.

Some RecA-like proteins with mutations at the MAW motif are reported in the literature. RecA13 (L51F) and RecA56 (R60C) display a null phenotype *in vivo* and are deficient in all activities, except ssDNA binding [17,18]. RecA (S44L), also characterized *in vivo*, acts like the wild-type RecA protein, but it lacks interaction with the *E*. *coli* UmuD'C complex [19]. RecA D48A and G54A are deficient in UV damage repair [20]. Within known mutants, only RecA S42A and RecA G55A behave completely like the wild-type RecA protein [21]. The *E*. *coli* RecA residue L47, of the MAW motif, is conserved in 95% of the RecA proteins [8,16], and is part of a group of non-polar residues of the MAW motif that forms the protein hydrophobic core together with L51, I61 and I64 [6,8,11,12,14,15,22,23]. Despite Leucine 47 substitution by Tryptophan was considered to be the most preferable since the latter is the only aromatic residue sufficiently large to enhance DNA binding, *E*. *coli* RecA L47W did not show higher ability to induce LexA auto-cleavage than the wild type [9]. Notwithstanding the evaluation of some apolar-polar substitutions at MAW motif, its precise function still unknown.

In the present study, we report the characterization of the mutant RecA L53Q (that corresponds to *E*. *coli* mutant L47Q) protein of *H*. *seropedicae*. We used *in vitro* assays, such as ATP hydrolysis and DNA strand-exchange to evaluate the replacement effect of a very conserved apolar residue by a polar one. The Methyl methanesulfonate and Ultraviolet radiation assays were used to evaluate the survival of *E*. *coli* cells expressing the mutant RecA L53Q in response to mutagenic agents. Molecular Dynamics simulations were applied to the wild-type and RecA L53Q protein model, in order to get insights at a molecular level of the effects of the point mutation at the MAW motif.

## 2. Materials and methods

### 2.1 Plasmid construction, cloning and protein overexpression

The plasmid pAET-HMK [24], which contains the wild-type *recA* gene from *Herbaspirillum seropedicae* cloned into pET28b+HMK vector, was amplified using quickchange kit to insert the L53Q mutation, yielding the plasmid pAETL53Q-HMK.

*E*. *coli* strains BL21 (λDE3) pLyS or B834 (DE3) (Novagen) containing the plasmids pAET-HMK or pAETL53Q-HMK was grown in Terrific Broth medium on a rotary shaker to an OD_600_ of 0.3 at 37°C. At this point, the temperature was lowered to 25°C for 30 min and then 1 mM isopropyl-β-D-thiogalactopyranoside was added to induce the expression of the His-tagged wild-type *H*. *seropedicae* RecA (HsRecA) and mutant HsRecA L53Q. After an additional incubation for 3–4 h, cells were harvested by centrifugation and stored at -20°C. A purity of 96–98% with an overall yield of 88–100 mg/L of culture was achieved.

### 2.2 His-tagged wild-type HsRecA and HsRecA L53Q purification

All purification steps were carried out at 4°C. The frozen cells were thawed on ice and lysed in a cell disruptor (Constant Systems Ltd., UK) in 25–50 mL buffer A_NI_ (25 mM NaH_2_PO_4_ pH 7.0, 5% glycerol, and 0.5 M NaCl) containing a tablet of a cocktail of protease inhibitors. The soluble protein extract was loaded onto a 5 mL Hi-Trap Chelating column (GE Healthcare) charged as indicated by the manufacturer. After being submitted to an imidazole gradient (0.04–0.8 M) in buffer A_NI_, the fractions containing the HsRecA or the HsRecA L53Q proteins were dialyzed and stored at -80°C after freezing in liquid nitrogen.

### 2.3 ATP binding assay and UV crosslinking studies

ATP binding assays by UV crosslinking were performed as previously described [24]. Standard reaction mixtures (20 μL) containing ATP binding buffer (20 mM Tris-HCl pH 7.5, 20 mM potassium chloride and 10 mM magnesium chloride), 2.5 μM HsRecA or HsRecA L53Q, 6.5 μM circular φX174 ssDNA (New England Biolabs) and 37 kBq [α^32^P] ATP (111 TBq/mmol) were placed on a glass plate which was covered by a plastic film (ParafilmTM) and kept on ice. The reaction was then carried out with a UV lamp (254 nm, UVG-54; UVP, San Gabriel, CA, USA) at a distance of 3 cm for 20 min and quenched by the addition of 3 μL 6× SDS-PAGE loading dye. After heating at 95°C for 3 min, the samples were analyzed on SDS-PAGE gels (12.5%). Gels were stained with Coomassie Blue to detect the proteins and, after drying, the cross-linked products were visualized by a FLA-5000 PhosphorImager in which images were analyzed using the densitometry AIDA program. The *E*. *coli* wild-type RecA (EcRecA, GE Healthcare) was used as control.

### 2.4 ATPase assay

ATPase assays were performed as previously described [24]. The HsRecA and HsRecA L53Q proteins were incubated with 44 μM circular *φ*X174 ssDNA in ATPase buffer (25 mM Tris-acetate pH 7.5, 1 mM DTT, 3 mM potassium glutamate, 10 mM magnesium acetate and 5% (w/v) glycerol) at 37 ^o^C for 10 min. The reactions were initiated by the addition of SSB protein and labeled ATP, prepared by mixing 1.1 MBq [α^32^P]ATP (111 TBq/mmol), 50 μL of cold ATP (4 mM) and 97 μL of water (MilliQ). Reactions were stopped at different times (see figure legends for details) by the addition of 2 M cold formic acid (5V) and kept on ice. Aliquots of each reaction mixture were analyzed using thin layer chromatography as previously described [25]. The amounts of labeled ATP and ADP present in each reaction mixture were determined after imaging with a FLA-5000 PhosphorImager and using the densitometry AIDA program. The EcRecA (GE Healthcare) was used as control.

### 2.5 DNA strand exchange assay

The DNA strand assay was adapted from Drees et al. (2004) [26], as previously described [24]. The RecA protein (6.7 μM) was pre-incubated with 22.6 μM circular *φ*X174 ssDNA and an ATP regenerating system [10 units mL^-1^ pyruvate kinase (Sigma) and 5 mmol phosphoenolpyruvate (Sigma)] for 10 min in 20 μL of buffer E (25 mM Tris-acetate pH 7.5, 1 mM DTT, 5% (w/v) glycerol, 3 mM potassium glutamate and 10 mM magnesium acetate) at 37 ^o^C. SSB protein (2 μM) and ATP (3 mM) were then added, followed by another 6 min of incubation. The reactions were initiated by the addition of 15.6 μM linear *φ*X174 dsDNA and stopped after 90 min by the addition of phenol/SDS 10% (2:1) solution. The samples were electrophoresed in 0.8% agarose gels in TAE buffer, stained with ethydium bromide and exposed to ultraviolet light. The EcRecA (GE Healthcare) was used as control.

### 2.6 MMS and UV sensitivity assay

*E*. *coli* XL1 Blue (*recA1 endA1 gyrA96 thi-1 hsdR17 supE44 relA1 lac* [F´ *proAB lacI*q ZΔ*M15 Tn10* (Tetr)] [27], was transformed with the plasmids pBMR503 [28] or pBMR503-L53Q, which express the wild-type HsRecA and mutant HsRecA L53Q by its own promoter, respectively. pBMR503-L53Q was obtained by using pBMR503 as template, L53Q-F 5´-GCTCACTGGGCC**A**GGACATCGCCCTG-3´ and L53Q-R 5´-CAGGGCGATGTCC**T**GGCCCAGTGAGC-3´ as primers and the QuikChange Site Directed Mutagenesis kit (Stratagene). The bold nucleotides indicate the ones modified from the template plasmid.

The sensitivity to MMS and UV of *E*. *coli* XL1 Blue with or without the plasmids of interest was determined as previously described [20]. Cells were grown on LB medium agar plates [29] containing increasing concentrations of MMS (0.0–2 mM) at 30°C. UV was delivered by a germicidal bulb (254 nm, radiation of 0.3 mJcm^-2^ s^-1^, Spectronics Corporation, Westbury, NY) at a distance of 15 cm in the absence of daylight illumination. After 12, 24 and 36 h incubation, the number of viable colonies was determined using the microdrop method of Miles and Misra [30]. *E*. *coli* 7118 (*supE thi* Δ(*lac-proAB*) F' [*proAB*+ *lacI*^q^ *lacZ* ΔM15], [31]) was used as positive control. The results are the average of two independent experiments in quadruplicate.

### 2.7 Protein model building and molecular dynamics

The HsRecA crystallographic model lacks regions for which electron density was not observed (amino acid ranges 1–5, 41–43, 164–175, 201–218 and 343–351) [15] and the molecule is in the collapsed, therefore inactive, conformation. Initially, the first 4 missing ranges were modeled for a single monomer based upon EcRecA structures (monomer C of PDB entries 3CMU and 3CMT), with the MODELLER homology modeling package [32,33] using a structural alignment for these three structures performed with TCOFFEE [34]. This was necessary for the correct positioning of residues preceding ALA45 (N-terminal), required for RecA filament formation. The segments of the residue ranges 164–175 and 201–218 (those involved in DNA contacts) were also derived from these templates. The best model was chosen according to a DOPE score assessment [35]. The last segment (residue range 343–351) was generated using the I-TASSER Server [36], which automatically selected as templates the structures of EcRecA (PDB 3CMU, 3CMV and 3CMW), *Thermotoga maritima* (PDB 3HR8), *Mycobacterium smegmatis* (PDB 1UBG) and *Herbaspirillum seropedicae* (PDB 5JRJ) itself. Therefore, atom coordinates for most amino acids and the ATP molecules were taken from PDB entry 5JRJ, while its missing segments were modeled with either MODELLER or I-Tasser as described above.

The monomer thus built was used to generate a hexamer to present the extended, therefore active, conformation model of hexameric HsRecA. Each monomer was superposed to its homologue in the hexameric form of EcRecA (code 3CMU). Then, ligand dsDNA (composition (5'-D(DTP_5_*DCP_3_*DAP*DCP_2_*DTP_3_*DT)-3') and (5'-D(P*DGP_2_*DTP*DGP_2_*DG)-3')) was included from the superposition of this model to each monomer of EcRecA (PDB 3CMT) using PYMOL [37], to give the complex HsRecA-ATP-dsDNA hexamer.

This final model, thus obtained, was used to get the mutant L54Q structure; both structures were then submitted to MD simulations. All system preparation, equilibration and productive steps for MD simulations were accomplished using modules belonging to AmberTools17 and Amber16 simulation packages [38]. The structures were solvated with TIP3P water model molecules in a box whose edges were at least 11 Å far from any model atom using LeaP, which also performed the charge neutralization of the system. The MD protocol was as follows: two energy minimization steps (one using strong restrictions for all non-solvent atoms and the other allowing the whole system energy to minimize); dynamics for 50 ps to heat the system from 0 to 300 K with weak restrictions; dynamics for 50 ps with the same restrictions at constant temperature; dynamics for 60 ps to change from constant volume to constant pressure and, finally, a productive dynamics for 20 ns. All simulations were carried out in triplicates (each one with a different random seed) using the gpu version of PMEMD [39]. The trajectories were analyzed with CPPTRAJ [40].

## 3. Results

### 3.1 The L53 residue in MAW motif of RecA is not required for ATP binding

**Fig 1:**
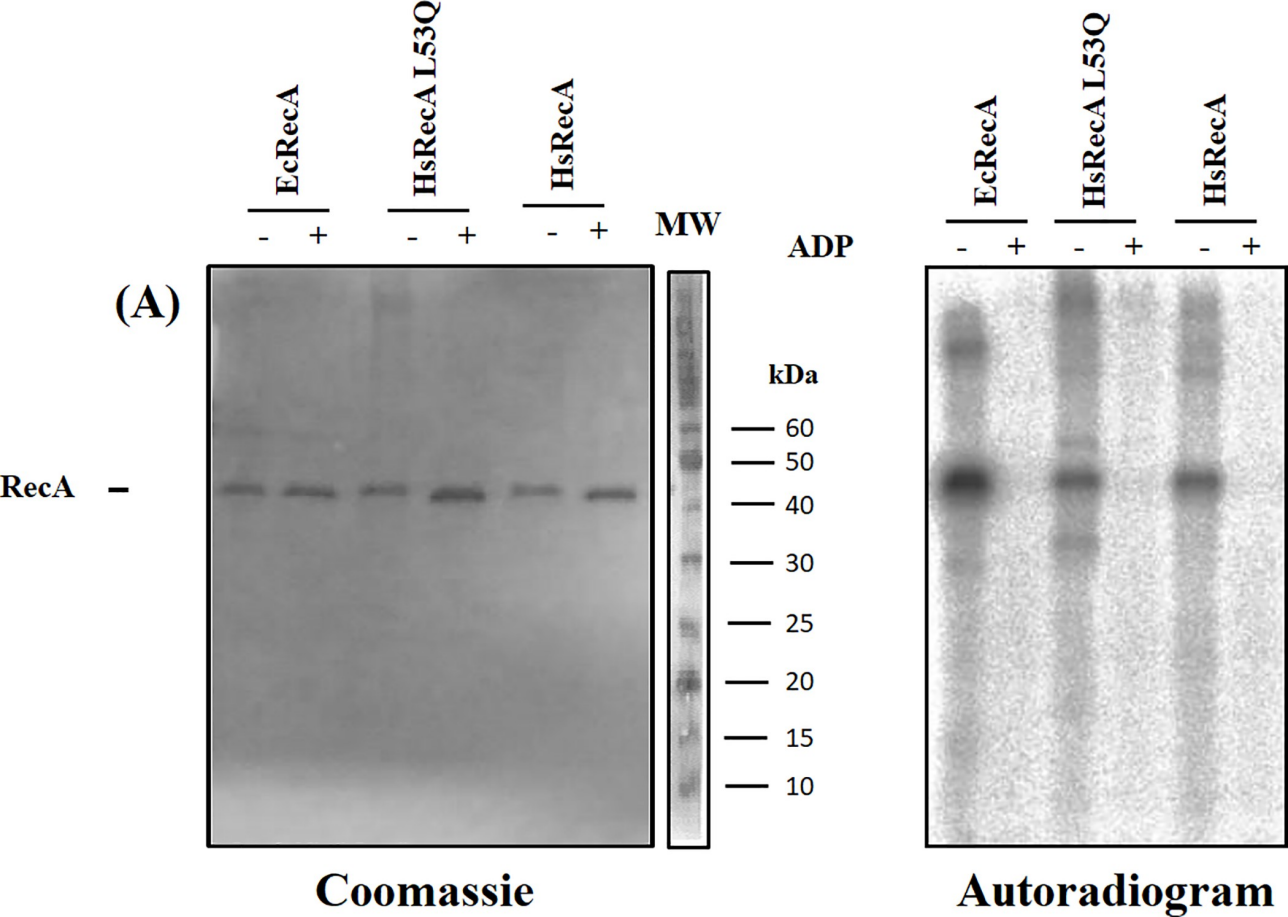
Effect of L53Q substitution in the ATP binding activity of H. seropedicae RecA protein. Legends: - and + indicate absence and presence of ADP, respectively. (A) Coomassie blue-stained SDS-PAGE gel. The separated lane at the right side of Fig 1A shows the molecular weight (MW) markers. (B) Autoradiography of [α^32^P] ATP-RecA labeled.

The ability of the HsRecA L53Q to bind ATP in the presence of ssDNA was confirmed and compared with the wild-type HsRecA and EcRecA. The [α^32^P]ATP—HsRecA L53Q complex was detected, and EcRecA, HsRecA and HsRecA L53Q remain covalently bound to [α^32^P]ATP after UV exposure (Fig 1B). In absence of ADP, band intensity for the products [α^32^P]ATP-HsRecA and [α^32^P]ATP-HsRecA L53Q was observed to be similar. This result shows that the substitution L53Q in MAW does not prevent ATP-binding on HsRecA protein. Moreover, when ADP is present in a concentration higher than that of ATP, no bands are observed for any of the RecA proteins studied, indicating that the binding of [α^32^P]ATP is specific to the ATP-binding site and is preserved in all proteins, including HsRecA L53Q.

### 3.2 The L53Q residue mutation in the MAW motif of HsRecA prevents ATP hydrolysis and strand exchange, but not the ssDNA-binding

Since RecA is a DNA-dependent ATPase [41], DNA binding can be indirectly measured using ATP hydrolysis. This assay was performed to check whether HsRecA L53Q can hydrolyze ATP and bind to ssDNA as the wild-type HsRecA does, as previously characterized [15,24].

**Fig 2:**
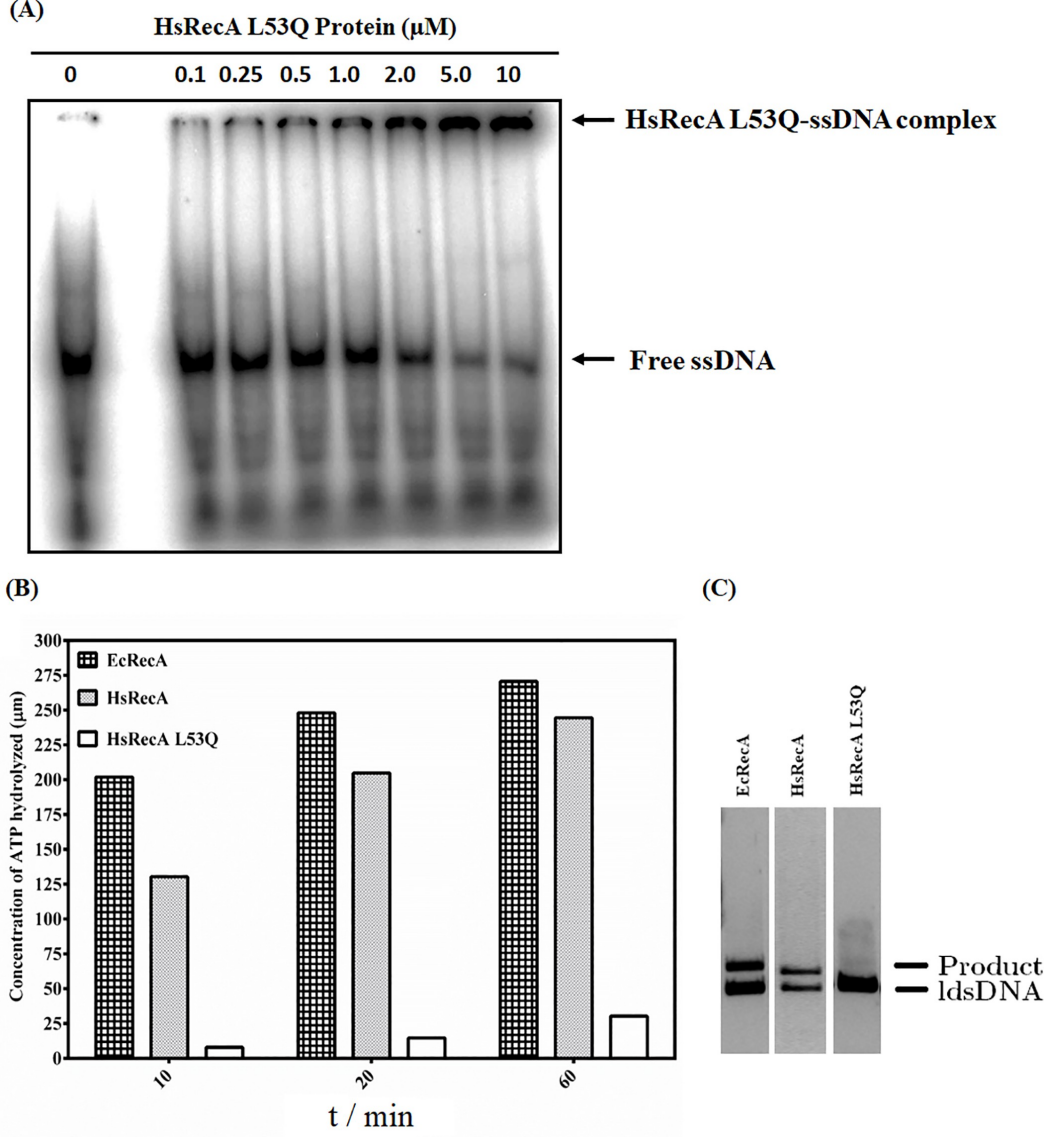
ssDNA-binding of HsRecA L53Q, ATPase and DNA strand exchange activity promoted by EcRecA, wild-type HsRecA and HsRecA L53Q proteins. (A) ssDNA-binding capacity of HsRecA L53Q assayed by using [α^32^P]-ssDNA 88 *mers*. (B) Amounts of ATP hydrolyzed. The ATPase activity profile of wild-type HsRecA was previously characterized [15,24]. (C) The three DNA strand exchange activity. These results are representative of three separate experiments.

The electrophoretic mobility shift assays were performed to confirm the ssDNA-binding of HsRecA L53 protein (Fig 2A). The assay confirmed that HsRecA L53Q has ssDNA-binding capacity as does the wild-type HsRecA protein [15]. The ATP hydrolysis rates of EcRecA, HsRecA and HsRecA L53Q were determined by measuring the concentration of labeled ATP hydrolyzed to ADP (Fig 2B). However, HsRecA L53Q does not show significant amounts of hydrolyzed ATP even bonded to ssDNA, or even after 60 min assayed.

The DNA-strand exchange promoted by EcRecA protein normally requires the presence of ATP. The ATP hydrolysis is required for the structural transition of the RecA protein-ssDNA complex, when RecA filament adopts an extended active conformation. However, the hydrolysis is not required for DNA strand exchange [42]. HsRecA L53Q is unable to promote DNA-strand exchange (Fig 2C) and it is likely unable to adopt the RecA active conformation, even in the presence of the bonded ssDNA and ATP.

### 3.3 Sensitivity of the mutant L53Q to MMS and UV irradiation

**Fig 3:**
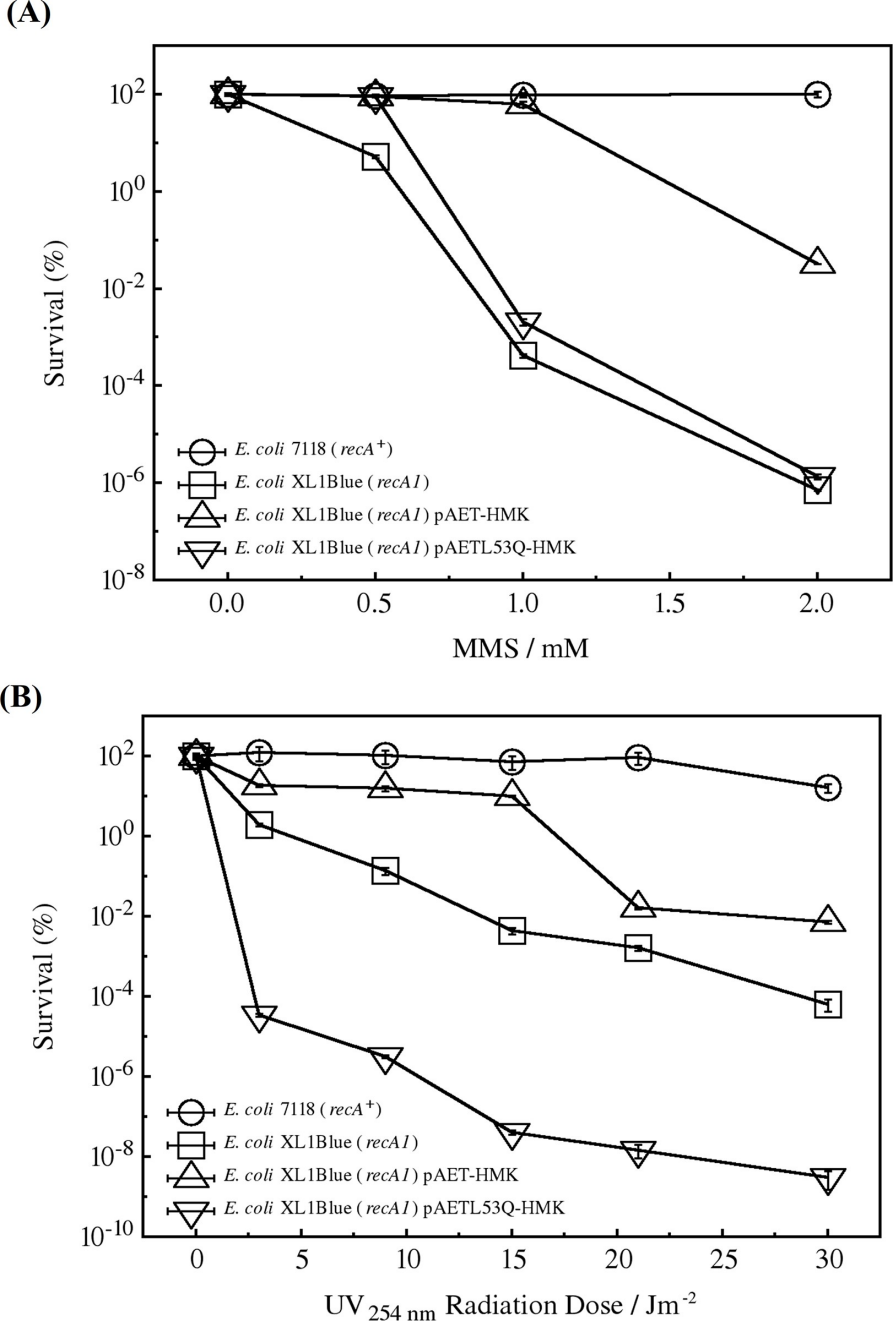
MMS and UV sensitivity of *E*. *coli* XL1Blue (*recA1*) expressing the wild-type HsRecA or the mutant HsRecA L53Q. (○) *E*. *coli* 7118 (*recA*+), (□) *E*. *coli* XL1Blue (*recA1*), and *E*. *coli* XL1Blue (*recA*1) harboring (Δ) pBMR503 (which encodes the wild-type HsRecA) or (∇) pBMR503-L53Q (which encodes the mutant HsRecA L53Q) were challenged with increasing concentration of MMS (A) and doses (time of exposition) of UV radiation (B). Each data point represents mean + SD. The results are the average of two independent experiments in quadruplicate.

We studied the effects of mutation L53Q on HsRecA in the DNA repair function by inserting either the wild-type HsRecA or the mutant HsRecA L53Q into the *E*. *coli* XL1Blue *recA1* strain and challenging it with the mutagenic inductors MMS and UV radiation (Fig 3A and 3B, respectively). *E*. *coli* 7118 (*recA*^+^) and *E*. *coli* XL1Blue (*recA1*) without any plasmid were used as a positive and negative controls, respectively.

*E*. *coli* 7118 (*recA*^+^) showed a maximum survival rate within all MMS tested concentrations (0.0–2 mM) (Fig 3A). On the other hand, *E*. *coli* XL1Blue (*recA1*) presented significant sensitivity even at the concentration of 0.5 mM MMS, such that at 2.0 mM MMS there was no survival. The decrease in the survival rate presented by the *E*. *coli* XL1Blue (*recA1*) strain demonstrates that DNA lesions caused by MMS were not effectively repaired and the homologues recombination pathway was negatively affected. That was already expected, since the mutant G160D RecA protein encoded by *E*. *coli* XL1Blue (*recA1)* does not exhibit DNA-strand exchange activity [17]. Nevertheless, the insertion of the pBMR503 plasmid into *E*. *coli* XL1Blue (*recA1*) induced a maximal and constant survival rate up to 1 mM MMS and partially recovered it in the presence of 2 mM MMS. This indicates that the heterologous expression of the wild-type HsRecA almost completely repaired the DNA damage caused by MMS. However, the expression of the mutant HsRecA L53Q, from the pBMR503-L53Q plasmid, in this strain could not promote DNA repair; it presented 83.03% of survival rate at a 0.5 mM MMS and none at 2.0 mM MMS.

UV irradiation also presented extreme lethality to *E*. *coli* XL1Blue *recA*^-^ strain: after UV dose of 3 Jm^-2^ of irradiation, the survival rate decreased to less than 1.0% and with more 15 Jm^-2^, only 0.4% of the cells survived (Fig 3B). In contrast, *E*. *coli* 7118 (*recA*^+^) presented a high survival rate in all of the tested irradiation times. Wild-type HsRecA partially restored the ability to repair DNA damage caused by UV in *E*. *coli XL1Blue (recA1)* by the nucleotide excision repair pathway, photolyases and DNA glycosylase, but not in the HsRecA L43Q. In fact, it presented the lowest capacity to repair DNA, as the survival rate dropped down to 0.1% yet at only 3 Jm^-2^ of UV exposition.

### 3.4 Molecular dynamics studies

**Fig 4:**
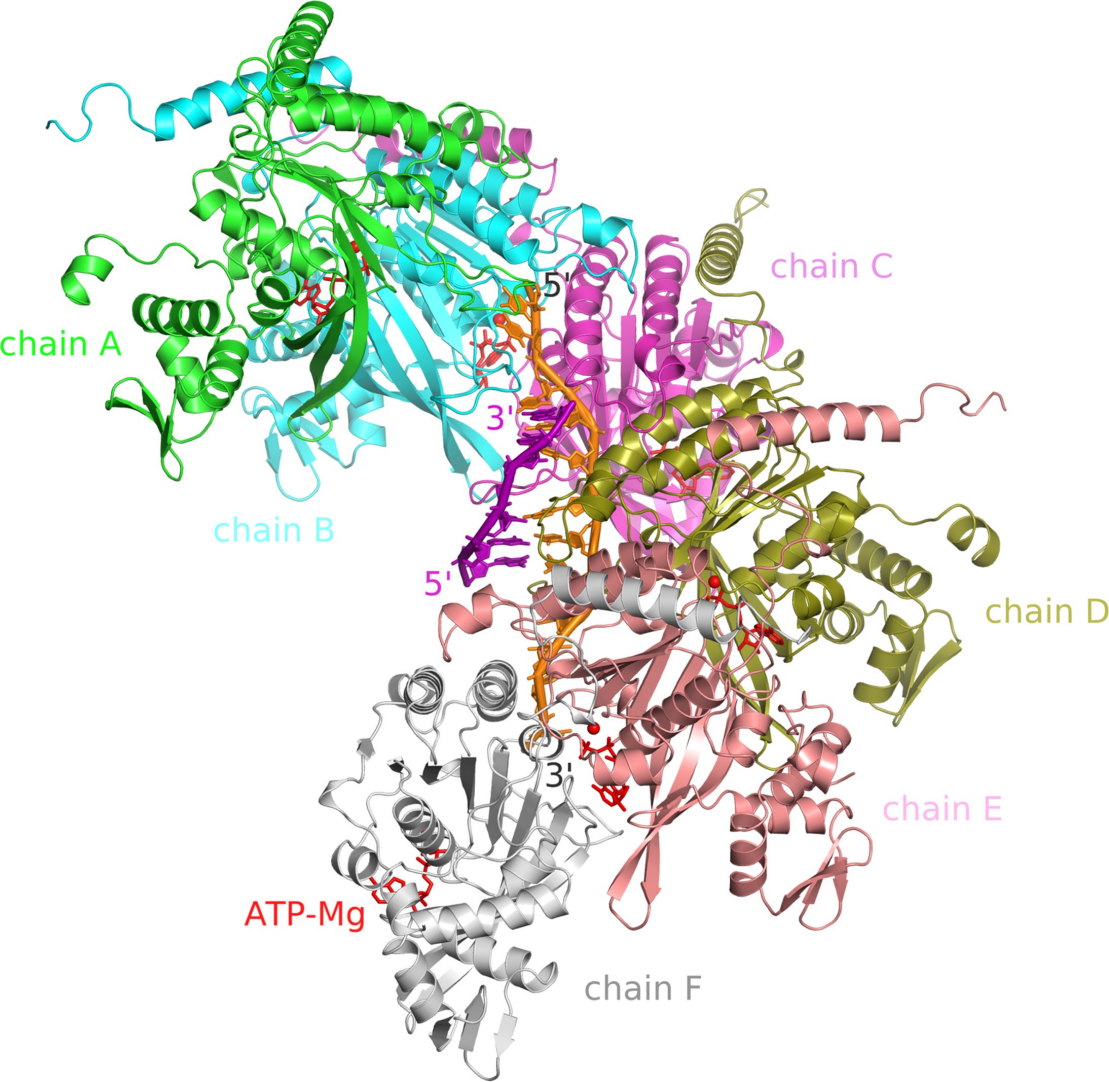
Model for HsRecA-ATP-dsDNA hexamer. The complete model of HsRecA hexamer (shown in cartoons, one different color for each monomer) containing dsDNA (shown as orange and purple sticks), ATP (shown as red sticks) and magnesium ions (shown as red spheres).

The hexameric HsRecA-ATP-dsDNA model produced from the HsRecA crystallographic structure, complemented with the missing regions and tailored to the active conformation is shown in Fig 4.

**Fig 5:**
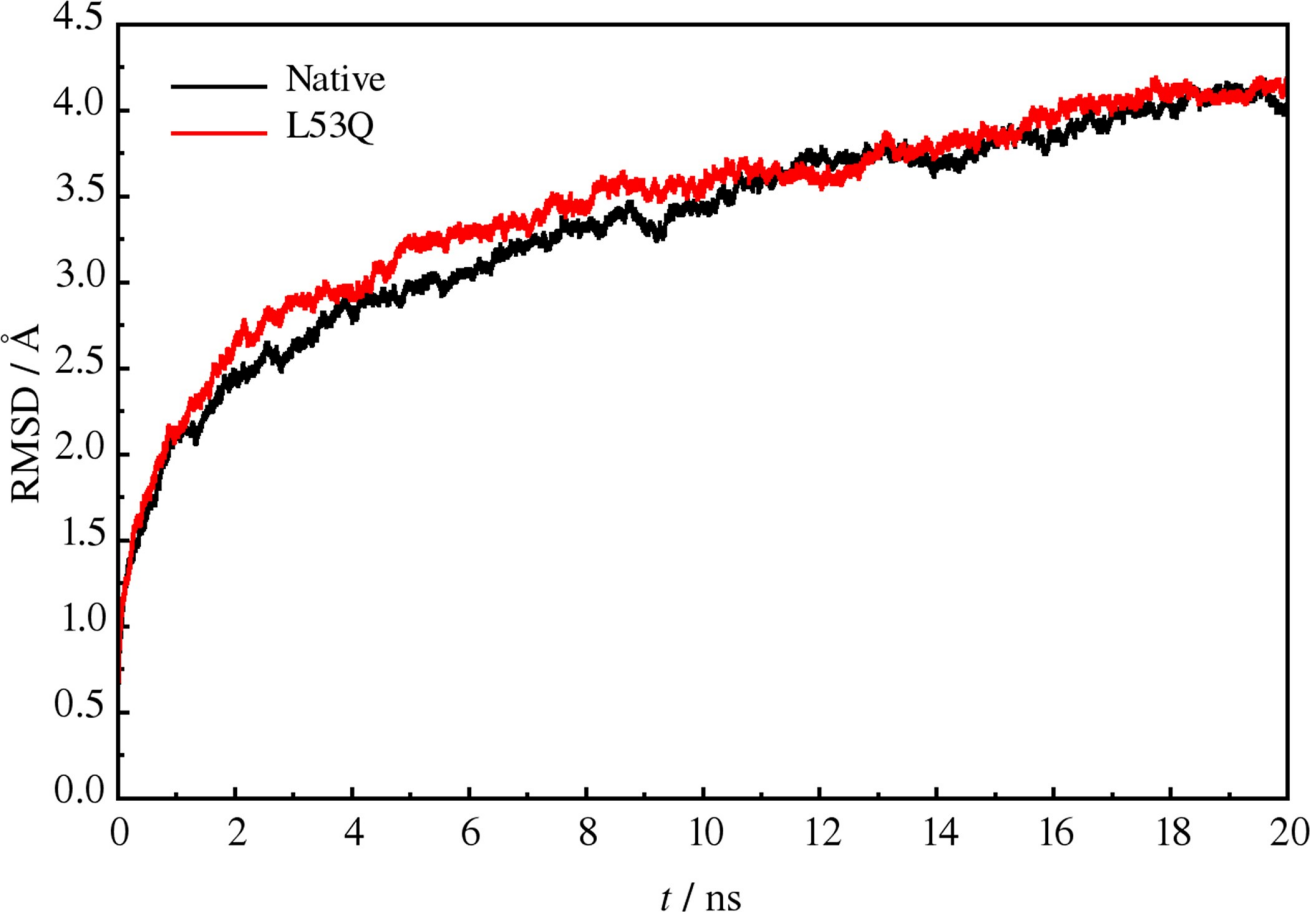
Average root mean square deviation plot. The black and red tracings are for wild-type and L53Q structures, respectively. Around 18 ns both structures seem to become stable.

To assess the structural stability in each MD trajectory, the root-mean-square deviations (RMSDs) of the Cα atoms were calculated from the trajectories over the whole 20 ns interval using the initial structure as a reference (Fig 5, which shows the averages among chains and replicates). In this simulation, deviations reached an apparent stability plateau around 4 Å after 18 ns. In addition, the overall behaviors of either the wild-type or the mutant protein are similar during the MD simulations, indicating that the L53Q mutation did not cause large structural instability in the HsRecA structure, as previously observed in other point mutation studies [43]. As a matter of fact, binding capacity to ATP and DNA is not changed, as observed in other studies, but the catalytic process, which must comprehend conformational changes at the MAW motif region, is impaired [44].

**Fig 6:**
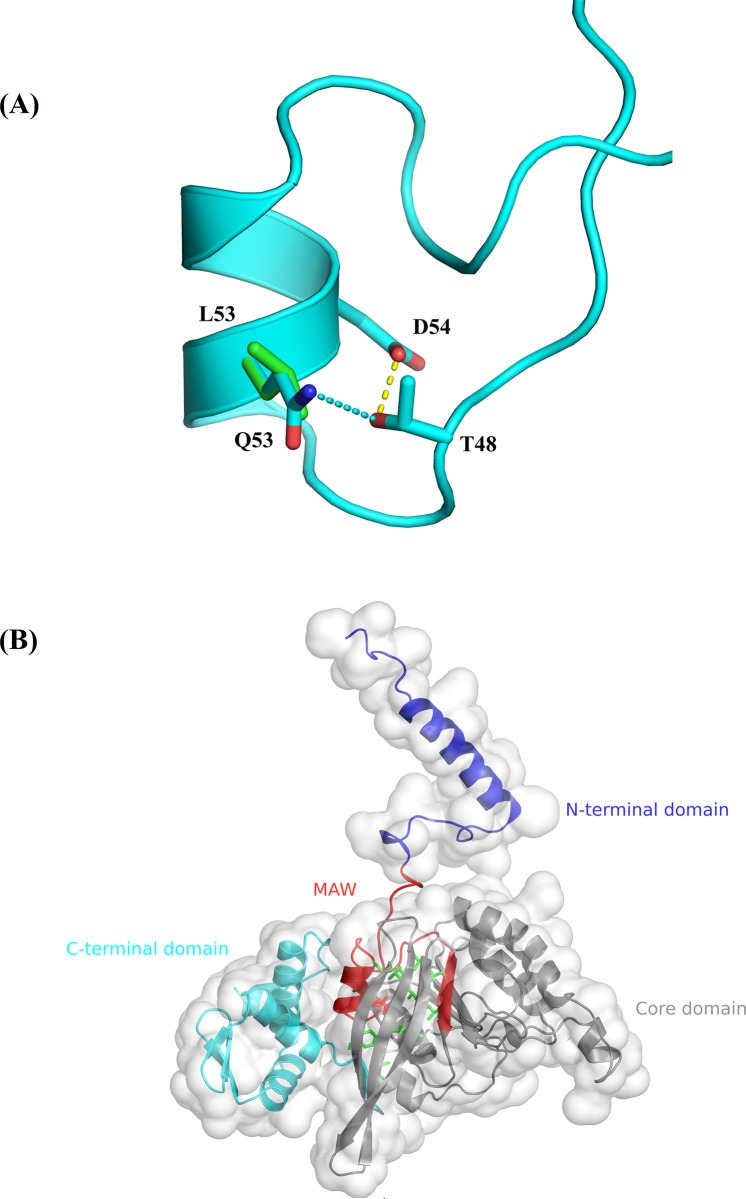
Residue 53 environment in HsRecA structure. (A) Superposition of the local view of the MAW motif, with residue side chains drawn as sticks–green for the wild type and cyan for the L53Q). The cyan dashed line shows the hydrogen bond that connects Q53:NE2 to T48:OG1 (present only in the mutant structure), the yellow dashed line shows hydrogen bonds that connect T48:OG1 to D54:OD1 (present in both wild-type and mutant structures). (B) Overall view, the MAW motif is shown in red (sticks shown for Gln53 side chain), buried within the hydrophobic core (sticks shown in green for residue side chains). The N-terminal and C-terminal domains are shown in blue and cyan, respectively. The structure is surrounded by the solvent accessible surface, except for the MAW motif.

From the MD simulations, we observed that during most part of the time (average 86.12%), in all replicates and monomers analyzed, the extra (polarity conferring) NE2 of the Q53 residue (part of the MAW motif and of the hydrophobic core) in the mutant forms a hydrogen bond mainly with the OG1 of the T48 residue. Such interaction confers the MAW motif other movement effect apart from the hydrophobic core in the HsRecA structure (Fig 6A). Additionally, T48 forms a hydrogen bond with D54 (through OD) in both wild-type and mutant structures; in the latter, therefore, an indirect bond between Q53 and D54 side chains.

**Fig 7:**
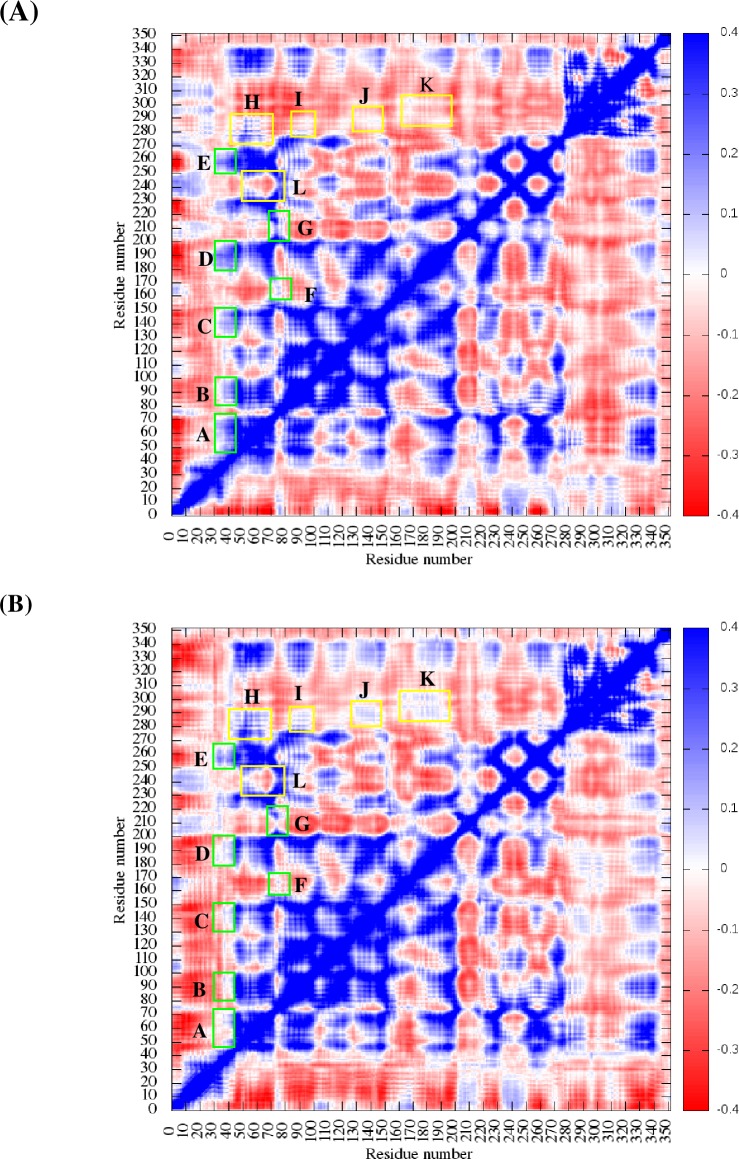
Intrachain cross correlation maps. Plot for (A) the native structure and (B) the L53Q mutant, areas with decreased and increased correlation are highlighted with green and yellow rectangles, respectively.

Intrachain dynamic cross correlation maps of the Cα‐atoms, averaged among the monomers and replicates, are shown for the wild-type and mutant structures in Fig 7A and 7B, respectively, over the 20 ns of MD simulations. Some differences of the dynamical movement correlations along residues 30–40 (residues of MAW motif) were detected and highlighted by green rectangles (labeled from A to E) in Fig 7B. Moreover, residues E74-T79, involved in ATP contacts, residues that form the DNA binding site, residue F210 and G167, that form Loop L2 (rectangle F) and Loop L1 (rectangle G), respectively, also presented differences. Yet, other noteworthy feature is the apparent initial difference of dynamical correlations along the region spanning residue ranges 40–70 and 230–250 (yellow rectangles, labeled L), which decreased and even inverted the correlation values. On the other hand, residues of the ATPase core and the RecA C-terminal domain (also highlighted with yellow rectangles, labelled H, I, J and K) increased positive dynamical correlations.

**Fig 8:**
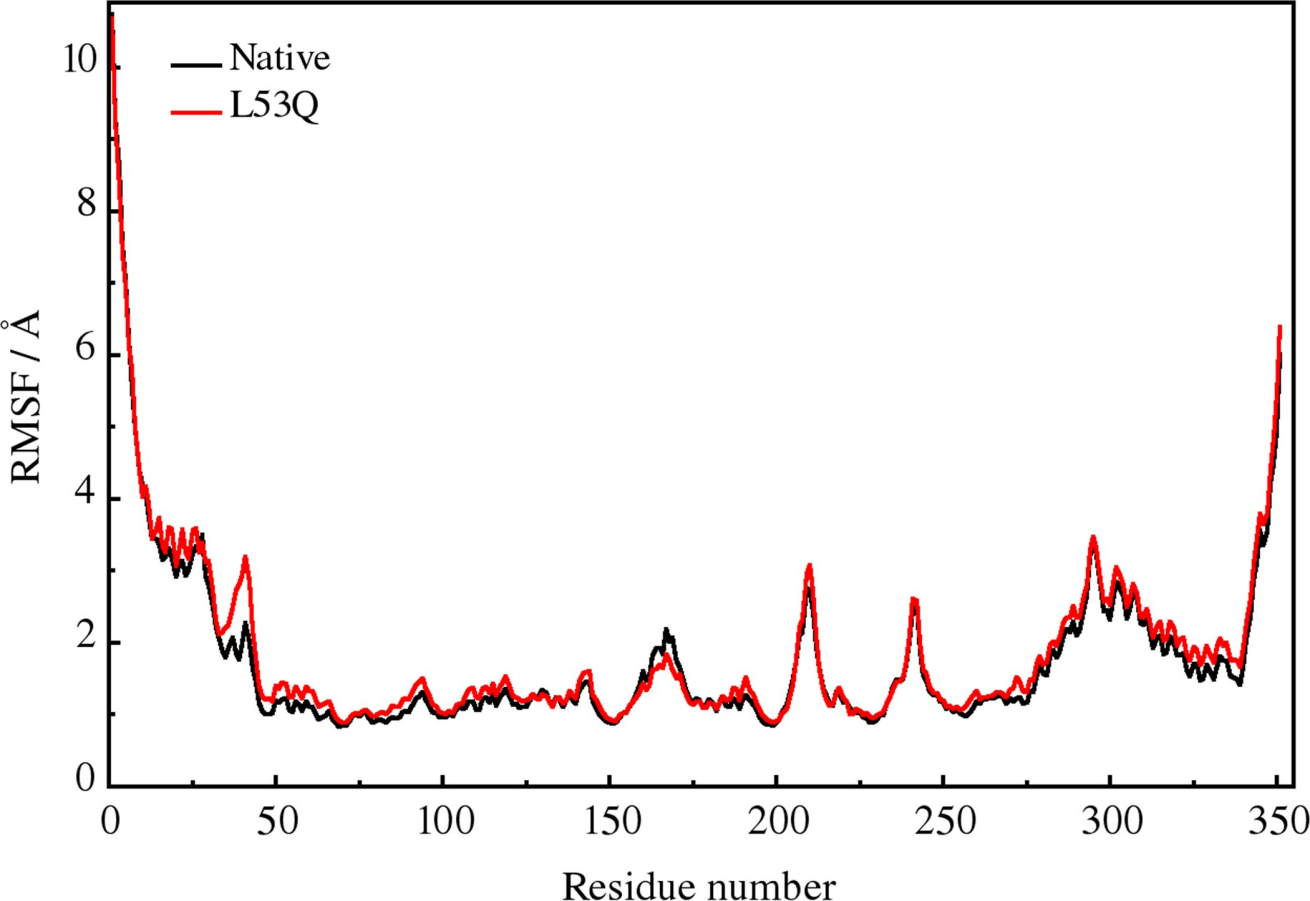
Averages of the RMS fluctuations per residue for the Cαatoms during 20 ns MD simulation.

Fig 8 shows averaged RMS fluctuations for each residue Cαatoms during 20 ns MD simulation. For most of the residues, the RMS fluctuation of the HsRecA L53Q is slightly larger than that of the HsRecA, with average values of 1.75 Å for the native protein and 1.87 Å for the L53Q mutant. Nevertheless, a larger difference is observed between residues 34–44 which comprehend the stretch right before helix 2 that harbours the mutation.

### 3.5 Discussion

In the present work, we report the biochemical activity and structural effect of the L53Q mutation at the MAW motif of the RecA protein from *H*. *seropedicae*. Despite its importance, the “Make ATP Work” (MAW) motif was only briefly explored in previous RecA characterizations [9,16].

The HsRecA wild-type and L53Q proteins were overexpressed from the pET28a plasmids and then purified. Their activities were evaluated by *in vitro* and *in vivo* assays and their structures were analyzed *in silico*.

The cross-linked complexes [α^32^P]ATP–HsRecA wild-type and [α^32^P]ATP–HsRecA L53Q detected in ATP binding assays indicated that the MAW motif does not control the nucleotide binding capacity of RecA protein (Fig 1A and 1B). Hence, electrophoretic mobility shift assays confirmed that HsRecA L53Q forms complexes with ssDNA (Fig 2A), as was previously shown for the wild-type HsRecA [15]. This finding suggests that the L53Q mutation does not disrupt the DNA and ATP binding capacity. On the other hand, it impairs ATP hydrolysis and DNA strand exchange (Fig 2B and 2C). It has been proposed that the MAW motif modulates interactions with DNA and acts as a conformation switch that responds to different cofactors [8,16].

The performed ATPase and DNA binding assays indicated that the dynamics of structural conformation transition adopted by the MAW motif during the catalytic process is essential for the RecA activity, but the mutation does not the affect the plain binding of either ATP, ADP or ssDNA (Figs 1 and 2A). Despite the fact that HsRecA L53Q protein expression by T7 promoter is higher than the endogenously chromosomal expression of wild-type EcRecA protein by its own promoter in the expression system used (*E*. *coli* strains BL21 (λDE3) pLyS or B834 (DE3)) and the fact that an affinity column was used in the purification, we cannot rule out the presence of the wild-type EcRecA protein in the final purified fractions, even more than a purity > 95% detected by SDS-PAGE gel. Additionally, these proteins with similar molecular weight cannot be distinguished on denaturing gel. It could explain the low ATP hydrolysis rates increase (Fig 2B), and the presence of intermediate molecules traces in the DNA strand exchange reaction (Fig 2C, right lane). Britt et al. (2011) reported the interference of an inactive RecA (EcRecA K72R) on wild-type EcRecA activities and showed that the level of interference is proportional to the amount of both proteins [45].

The RecA proteins of *Mycoplasma pulmonis*, *Myxococcus xanthus* and *Thermotoga maritima* do not present a Leucine at the corresponding position (53) in *H*. *seropedicae*, however, they present other hydrophobic amino acid, either isoleucine or valine, what preserves their functionalities [8]. In *E*. *coli*, the replacement R60C (in MAW motif), creates a protein unable to perform the DNA-strand exchange [17]. Residue R60 has been proposed to be involved in the communication between monomers [8]. The mutant *E*. *coli* RecA S44L, also characterized *in vivo*, acts like the wild-type RecA, but lacks interaction with the *E*. *coli* UmuD'C complex [19]. The double mutant D234A/G240A of the RecA-like protein of *Ustilago maydis* showed defects in DNA repair [20]. These mutations correspond to D48A and G54A in the *E*. *coli* RecA protein, respectively.

To determine the effects of the L53Q mutation on cell survival, the expression of either HsRecA L53Q or wild-type protein was induced in *E*. *coli* XL1Blue (*recA1*) from its own promoter, and the cells were challenged with Methyl methanesulfonate (MMS) and Ultraviolet radiation (UV), agents that induce the SOS response.

MMS generates alkyl adducts, such as the N^3^-methyladenine (N^3^-mdA) minor groove lesions [46]. DNA damage caused by alkylating agents is predominantly repaired by the base excision repair pathway and DNA alkyltransferases [47]. Recently, Thrall et al. (2017) revealed the participation of PolIV and PolV into MMS lesion repair by single-molecule imaging. MMS treatment led to the wild-type EcRecA cellular reorganization, with RecA foci moving to the same midcell position as PolIV and SSB, near the replication fork and persistent ssDNA tracts. PolV also carried out some DNA synthesis under these conditions without competing directly with PolIV for binding sites [46]. *E*. *coli recA*^+^ showed the highest survival rate (Fig 3A) because many repair mechanisms were active, like base excision repair, homologous recombination repair, and mutagenic repair.

*E*. *coli recA1* showed the lowest survival rate at the MMS challenge. Its G160D mutation impairs the RecA bundle formation, that is, the single extended RecA nucleoprotein filaments [48] and therefore unable SOS induction and homologous recombination function [49]. *E*. *coli recA1* survival rate was almost completely recovered by the expression of wild-type HsRecA. Possibly, the recovery was not complete because of the dynamical interference between EcRecA G160D and HsRecA proteins, expressed from the host chromosomal and plasmid genes, respectively. In *E*. *coli* cells harboring the pBMR503 plasmid (to express wild-type HsRecA), both RecA proteins can bind ssDNA fragments and form nucleofilaments, reducing DNA repair efficiency. Horii et al. (1992) also reported the interference of the N and C–terminus truncated RecAs expression, even in a *recA*^*+*^ context of the *E*. *coli* cell [50].

*E*. *coli recA1*, without any extra protein expression or with the expression of HsRecA L53Q, showed an equivalent low survival rates (Fig 3A). The SOS response might have been induced in all tested cells since all RecA proteins (EcRecA, EcRecA G160D, HsRecA, HsRecA L53Q) are able to bind to ssDNA and to form the extended nucleofilaments that catalyze LexA auto-cleavage. This reaction is catalyzed by an activated form of RecA, that acts as a co-protease to stimulate the self-cleavage activity of LexA. Therefore, all SOS regulon genes were expressed, including the translesion synthesis polymerases PolIV and PolV coding genes. So, PolIV and PolV repair might have occurred in *E*. *coli recA1* cells with or without HsRecA L53Q, since a mutant RecA without the ability to break ATP can still activate DNA polymerase V to breakdown this molecule itself and therefore perform translesion synthesis (TLS) [51]. On the other hand, homologous recombination repair might not have occurred, since these mechanisms are dependent on RecA strand exchange activity. Based on the fact that Homologous Recombination repair requires the DNA strand exchange activity of RecA, cells that contained the mutant RecA (EcRecA G160D and/or HsRecA L53Q) presented the worst survival profile.

UV lesions are repaired by non-mutagenic mechanisms, like nucleotide excision repair, and they are bypassed by PolIV and PolV, which induce mutations. PolV accumulates later in the SOS response and acts upon more severe forms of DNA damage, including major groove lesions caused by UV radiation. In the present work, the tested bacteria presented a similar profile of sensitivity at either UV or MMS (Fig 3), and again the SOS response might have been induced in all tested cells in the presence of UV. However, due to the higher severity of the UV induced mutations, repair mechanisms might have been demanded more intensively in this condition. Based on the fact that neither recA56 (MAW mutant) or recA56 cells express RecA proteins able to form nucleoprotein active structures [48,52], which are essential to perform Homologous recombination and to induce SOS response, further analysis are required to explain the highest sensitivity to UV presented by *E*. *coli recA1* cell with HsRecA L53Q than without it.

Molecular dynamics was used to evaluate structural effects of the L53Q mutation in HsRecA that could be related to its inability to hydrolyze ATP and to promote DNA strand exchange *in vitro*, two activities that restricted DNA repair in the presence of the mutagenic agents MMS and UV *in vivo*. The simulation results did not show any overall large conformational change in HsRecA L53Q structure, over a 20 ns timescale. Nevertheless, NE2 of the residue Q53 in the mutant HsRecA L53Q forms a hydrogen bond with the OG1 of T49 during the most part (average 82.16%) of the time. This additional hydrogen bond is hypothesized to confer a partially independent motion of the MAW motif from the hydrophobic core, of which L53 partakes. Additionally, we observed some differences of dynamical cross correlations between residues of the MAW motif and those involved in contacts with ATP at its binding site, and with regions that include the DNA binding sites. Since RecA protein is a DNA-dependent ATPase and the MAW motif constitutes the activation gate for ATPase activity, the mutation seems to disturb the switch function of this motif, which could be a reason for the activity loss of HsRecAL53Q.
